# Defining Telemedicine and Engaging Future Medical Practitioners. Comment on “Telemedicine in Germany During the COVID-19 Pandemic: Multi-Professional National Survey”

**DOI:** 10.2196/23363

**Published:** 2021-02-17

**Authors:** Neha Sadik, Reem Salman

**Affiliations:** 1 Barts and the London School of Medicine and Dentistry Queen Mary University of London London United Kingdom

**Keywords:** telemedicine, coronavirus, COVID-19, medical education

We read with interest the recently published paper by Peine et al [1] on the views of health professionals on telemedicine. By surveying health professionals from across a variety of clinical settings and demographics, interesting results were obtained illustrating high levels of acceptance of telemedicine among health professionals in Germany. Acquiring an accurate view of telemedicine has never been more important than during the COVID-19 pandemic, where the usage of telemedicine to communicate safely with patients has increased dramatically.

The authors have made use of the word telemedicine throughout the paper, without specifically defining its meaning in this context. In fact, a 2007 study found up to 104 different peer-reviewed definitions of telemedicine [2] or “eHealth” across many different studies. Thus, we ask the authors of the study to clarify what they meant by telemedicine and if this term was defined for all study participants. We support a definition that encompasses the integration of technology mediums into the delivery of more accessible, improved, and cost-effective health care. The use of telemedicine extends beyond its role as a communication medium; it can be a resource for both health care professionals and patients, an instrument for diagnosis and screening, or a device to globalize medicine.

We note that a strength demonstrated in the survey was the large number of participants and the variety of different health professionals included, making it highly representative of the overall German health professional body. However, now that telemedicine has been incorporated into our day-to-day practice of medicine, it is extremely important to explore specific challenges of telemedicine to individual specialties. For example, recent research has shown that it may be difficult to form a trusting patient-doctor relationship using technology, which may be especially impactful in psychiatry and mental health care [3]. For this reason, the results of this study may reveal additional insight if responses were stratified by specialty. By identifying particular problems, solutions pertaining to each specialty may be discovered. For example, virtual reality (VR) technology that replicates a doctor’s office may help to create an environment where a lasting doctor-patient relationship can be established.

In addition, although a variety of health professionals were surveyed, we feel the views of medical students and clinical tutors are clearly missing from this study. As the first generation to fully experience the intersection of telemedicine and medical education, the opinions of this group could shape the future of medicine and better prepare upcoming doctors for practice in the post–COVID-19 era. By surveying medical students, a demographic that is mostly proficient in technology, we can find more innovative usages of telemedicine for maximal benefit to both doctors and patients.

From this, we conclude that despite the numerous benefits of telemedicine in clinical practice, there is a multitude of challenges to overcome. Major investment into technological infrastructure and training seems to be an unavoidable expense for the advancement of telemedicine. As telehealth becomes an integral part of the new global health care system, we must strive to research and actively work toward its incorporation in health services for efficient patient care at all levels.

## Abbreviations

VR: virtual reality

## References

[1] Peine Arne, Paffenholz Pia, Martin Lukas, Dohmen Sandra, Marx Gernot, Loosen Sven H (2020). Telemedicine in Germany During the COVID-19 Pandemic: Multi-Professional National Survey. J Med Internet Res.

[2] Sood Sanjay, Mbarika Victor, Jugoo Shakhina, Dookhy Reena, Doarn Charles R, Prakash Nupur, Merrell Ronald C (2007). What is telemedicine? A collection of 104 peer-reviewed perspectives and theoretical underpinnings. Telemed J E Health.

[3] Hoffmann Mariell, Wensing Michel, Peters-Klimm Frank, Szecsenyi Joachim, Hartmann Mechthild, Friederich Hans-Christoph, Haun Markus W (2020). Perspectives of Psychotherapists and Psychiatrists on Mental Health Care Integration Within Primary Care Via Video Consultations: Qualitative Preimplementation Study. J Med Internet Res.

